# Aging Increases Enterocyte Damage during a 3-Hour Exposure to Very Hot and Dry Heat: A Preliminary Study

**DOI:** 10.3390/biology12081088

**Published:** 2023-08-04

**Authors:** Josh Foster, Zachary J. Mckenna, Whitley C. Atkins, Caitlin P. Jarrard, Craig G. Crandall

**Affiliations:** 1 Institute for Exercise and Environmental Medicine, Texas Health Presbyterian Hospital Dallas, The University of Texas Southwestern Medical Center, Dallas, TX 75231, USA; zacharymckenna@texashealth.org (Z.J.M.); whitleyatkins@texashealth.org (W.C.A.); caitlinjarrard@texashealth.org (C.P.J.); craigcrandall@texashealth.org (C.G.C.); 2 Centre for Human and Applied Physiological Sciences, Faculty of Life Sciences and Medicine, King’s College London, London SE1 1UL, UK

**Keywords:** heat, hyperthermia, age, aging, gastrointestinal, intestines, barrier function

## Abstract

**Simple Summary:**

Older age is associated with a greater risk of hospital visits and death during extreme heat events (heat waves). Previous research has identified that older people have reduced sweating and cardiovascular responses to heat compared with younger people, which could explain these increased health risks. However, prolonged heat exposure can also damage the gut lining, potentially resulting in microbes to ‘leak’ from the intestines into the blood circulation. If left untreated, this can cause widespread inflammation across the body which can be fatal. Although damage to the gut lining is well accepted to contribute to the heat stroke, no studies have determined whether older people could be more at risk of heat-related gastrointestinal lining damage. We exposed 16 young, and 16 older people to a 3-h heat exposure in a very hot and dry environment, and tested whether there was a group difference in blood markers of gut lining damage. We showed that gut lining damage occurred to a greater extent in older people, which in a real-world heat wave, has the potential to result in a greater release of microbes into the circulation.

**Abstract:**

Profound heat stress can damage the gastrointestinal barrier, leading to microbial translocation from the gut and subsequent systemic inflammation. Despite the greater vulnerability of older people to heat wave-related morbidity and mortality, it is unknown if age modulates gastrointestinal barrier damage and inflammation during heat stress. Therefore, the aim of this study was to determine if aging impacted enterocyte damage and systemic inflammatory responses to a 3-h exposure to very hot and dry (47 °C, 15% humidity) heat with accompanying activities of daily living (intermittent activity at 3 METS). Data from 16 young (age 21 to 39 years) and 16 older (age 65 to 76 years) humans were used to address this aim. In each group, log-transformed plasma concentrations of intestinal fatty acid binding protein (I-FABP_log_), interleukin-8 (IL-8_log_), and tissue factor (TF_log_) were assessed as indices of enterocyte damage, systemic inflammation, and blood coagulation, respectively, before and after the 3-h heat exposure. In the younger cohort, I-FABP_log_ concentration did not increase from pre to post heat exposure (*p* = 0.264, *d* = 0.20), although it was elevated in the older group (*p* = 0.014, *d* = 0.67). The magnitude of the increase in I-FABP_log_ was greater in the older participants (*p* = 0.084, *d* = 0.55). Across all participants, there was no correlation between the change in core temperature and the change in IFABP_log_. There was no change in IL-8_log_ in the younger group (*p* = 0.193, *d* = 0.23) following heat exposure, but we observed a decrease in IL-8_log_ in the older group (*p* = 0.047, *d* = 0.48). TF_log_ decreased in the younger group (*p* = 0.071, *d* = 0.41), but did not change in the older group (*p* = 0.193, *d* = 0.15). Our data indicate that I-FABP_log_ concentration (an index of enterocyte damage) is increased in older humans during a 3-h extreme heat exposure. Future studies should determine whether this marker reflects increased gastrointestinal barrier permeability in older individuals during heat exposure.

## 1. Introduction

Extreme heat events (heatwaves) have the potential to cause mass morbidity and mortality in humans [1,2]. While some regions have a low risk due to the widespread adoption of air conditioning, many regions, including most developed countries, are vulnerable. For example, temperature extremes in the United Kingdom (UK) are increasing faster than projected from only several years ago [3,4]; however, less than 5% of households in the UK are equipped with AC, and AC is still not installed in most new-build housing developments [5]. In the UK, fewer than thirty days of above average temperatures in the summer of 2022 caused upwards of 6000 excess deaths in those aged ≥ 70 years, with no excess deaths reported in those aged < 70 years [6]. Such high mortality rates are likely mirrored by large increases in hospital visits for heat-related disorders. For instance, there were over 15,000 excess hospital admissions during the 1995 heatwave in Chicago [7].

The etiology of why older people are especially vulnerable to heat is still debated [8,9,10]. One of the major complications that limits our understanding in humans is that the cause of death during a heat wave is based on ICD codes, rather than autopsy per se. Since a large proportion of heat wave-related deaths do not occur in hospitals [6], the specific code linked to an individual death is effectively a ‘best guess’. Based on our understanding of the cardiovascular adjustments to heat stress, it is commonly assumed that heatwave-related deaths in older people are of cardiovascular origin [11]. At rest, skin blood flow in adults is estimated to be ~250 mL/min, but can reach up to ~8 L/min during profound hyperthermia [12]. Such an increase in skin blood flow helps facilitate convective heat loss between the skin and environment and allows high volumes of blood to be cooled by sweat evaporation before returning to the body core [13]. Likely due to limitations of cardiovascular function associated with aging, older people are less likely to simultaneously perfuse the skin and vital organs, such as the brain and heart, possibly leading to an ischemic event, particularly in those with existing cardiovascular disease [11].

To facilitate increases in skin blood flow, the splanchnic vascular bed undergoes vasoconstriction during whole-body heating in young adults [14,15,16,17]. Furthermore, Minson et al. showed that splanchnic blood flow decreased by ~50% during hyperthermia in both young and older adults, although absolute splanchnic blood flow was lower in older adults [18]. While such responses are indispensable for thermoregulation, this transient decrease in oxygen delivery to the intestines has the potential to increase the production of reactive oxygen species (ROS) within enterocytes [19]. Elevated and prolonged ROS production can increase gastrointestinal permeability and allow the translocation of microbes from the intestines to the systemic circulation [19,20,21,22,23]. In primates, the prophylactic administration of an anti-endotoxin antibody caused a 100% increase in survival at core temperatures that were consistently lethal in control animals (i.e., ~43.5 °C) [24]. Although an even greater core temperature of 44 °C was lethal in all primates, an administration of anti-endotoxin increased survival time from 81 to 428 min in that work. In addition, the depletion of the gut flora with antibiotics prior to heating significantly reduces the pro-inflammatory response associated with heat stroke [25].

A role for microbial translocation as a cause of death in older people during heatwave is not well investigated, despite several strong lines of preliminary evidence. First, older individuals admitted to the ICU for heat stroke during the 2003 heatwave in France showed similar pro-inflammatory responses to those admitted for sepsis alone [26]. In that study, the extent of the inflammatory response was a strong predictor of mortality. Second, independent of heat stress, human intestinal biopsies demonstrate a large reduction in the expression of tight junction and adherens junction proteins in older subjects compared with younger, healthy subjects [27]. Third, animal data show clear age-related decreases in baseline intestinal barrier integrity [28] that may be related to microbiota dysbiosis [29], reductions in mucus barrier function [30], and reductions in Paneth cell function [31]. Overall, these studies suggest that microbial translocation related to reduced gastrointestinal barrier integrity is more likely to occur in older individuals.

The influence of aging on GI barrier damage during heat stress has not been studied. This lack of knowledge presents a significant gap in our understanding regarding the pathophysiology of heatwave-related mortality and morbidity in older people. Therefore, the aim of this study was to investigate whether aging impacts markers of gastrointestinal barrier damage, systemic inflammation, and blood coagulation during a simulated heat wave, as assessed through changes in plasma intestinal fatty acid binding protein (I-FABP), interleukin-8 (IL-8), and Tissue Factor (TF) concentrations, respectively. We hypothesized that aging would be associated with greater concentrations of I-FABP, IL-8, and Tissue Factor, compared with younger adults, during simulated heat wave exposure.

## 2. Methods

### 2.1. Ethical Approval

The Institutional Review Boards of the University of Texas Southwestern Medical Center and Texas Health Presbyterian Hospital Dallas approved the informed consent and study protocol (STU-2019-1759), which conformed to standards set forth in the Declaration of Helsinki. Signed informed consent was provided by all participants before participation in the study.

### 2.2. Participants

Participant characteristics are shown in Table 1. A total of 40 subjects participated in this study, although data from only 16 young (8 males, 8 females) and 16 older (8 males, 8 females) subjects were analyzed. Two subjects (one young and one older) were excluded for missing data on hematocrit and hemoglobin, which are required for the calculation of plasma volume [32] and thus plasma concentrations of blood markers after heat stress. One younger subject was excluded for having extremely high baseline concentrations of I-FABP (>4000 pg/mL) and IL-8 (>20 pg/mL) that are not representative of a healthy cohort [33,34]. Pre and post heat exposure venous blood samples were not obtained for the remaining five subjects.

### 2.3. Visit 1—Consent, Screening, and Metabolic Heat Production Test

During visit 1, participants provided their signed informed consent to participate in the study. Next, participants completed a medical history form and their blood pressure was measured to confirm they met the inclusion criteria (SBP ≤ 140, and DBP ≤ 90 mmHg). Participants with any diagnosed gastrointestinal diseases or disorders were not permitted to be enrolled in this project. Their heart rate and rhythm were subsequently confirmed to be normal using a 12-lead electrocardiogram, with interpretation from a board-certified cardiologist if any suspected abnormalities were detected (UT Southwestern Medical Center). Participants then completed a body composition assessment using dual-energy X-ray absorptiometry. Finally, in a normothermic environment (24 °C, 30% RH) we conducted a 15-min cycling test to determine the external workload (in Watts) that corresponded to 3 METS via the assessment of expired gases using indirect calorimetry (Parvo Medics True-One Metabolic Measurement System, Parvo Medics, Salt Lake City, UT, USA).

### 2.4. Visit 2—Pre Heat Exposure

**Figure 1:**
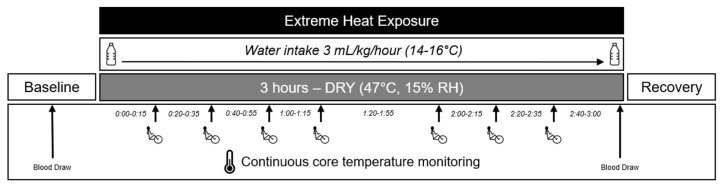
Experimental protocol. A venous blood sample was drawn following 30 min rest in a temperate environment. During the heat exposure, participants sat in the upright position throughout, and performed a 5 min bout of cycling exercise at 15, 35, 55, 75, 115, 135, and 155 min into the exposure. A second venous blood sample was drawn at the end of the 3 h exposure period while participants remained in the heat.

The experimental protocol is shown in Figure 1. To minimize the effects of diurnal variations, the arrival time for all experimental trials was between 08:00 and 10:00. Before each trial, participants were instructed to consume a light breakfast without caffeinated beverages. They were also prohibited from consuming alcohol and engaging in aerobic or resistance exercise for 24 h before testing. Upon arrival, participants provided a urine sample to confirm a euhydrated state, indicated by a urine specific gravity value of ≤1.020. Participants with a urine specific gravity value between 1.020 and 1.024 ingested 500 mL of water before testing. Next, venous blood draws were taken from the antecubital region. Core body temperature was measured using a rectal thermometer inserted 10 cm past the anal sphincter (Mon-a-therm, Mallinckrodt Medical, St. Louis, MO, USA). If participants did not consent to rectal temperature measurement, or if there were technical issues with the rectal thermocouple probe, core temperature was measured using an orally ingested telemetric pill (e-Celsius performance pill^®^, BodyCap©, Caen, France). Heart rate was measured from a 5-lead electrocardiogram (GE Medical Systems, Madison, WI, USA), and blood pressure was measured by automated auscultation of the brachial artery (Tango+, SunTech Medical, Morrisville, NC, USA).

Then, their nude body mass was assessed using a high-precision scale accurate to ±10 g (Mettler Toledo, OH, USA), and they were instrumented for the measurement of brachial blood pressure and skin temperature throughout the heat exposure.

### 2.5. Visit 2—Heat Exposure

Next, participants entered the environmental chamber set to the desired temperature of 47 °C (46.3 ± 0.6 °C) and 15% (17.3 ± 2%) RH. Males wore shorts and sports shoes, with the females also wearing a sports bra. After further instrumentation, which took up to 5 min, participants were seated in a semi-recumbent position on a chair with breathable fabric, followed by the onset of the 3-h heat exposure. Thermoregulatory (core temperature) and cardiovascular responses (blood pressure, heart rate) were monitored continuously. Participants consumed 3 mL/kg body mass of tap-temperature water every hour during the heat exposure to help maintain euhydration. A comprehensive assessment and comparison of the thermoregulatory responses between young and older adults is available in our companion manuscript [35].

### 2.6. Visit 2—Metabolic Heat Production

To mimic heat production associated with activities of daily living, participants performed seven 5-min bouts of leg cycling exercise at a light workload (3 METS) during the heat exposure. Cycling was performed in the seated upright position on a Monark 881E ergometer. The rate of metabolic heat production was confirmed during the first two 5-min bouts of exercise using indirect calorimetry (Parvo Medics True-One Metabolic Measurement System, Parvo Medics, Salt Lake City, UT, USA).

### 2.7. Venous Blood Draw and Assessment of Intestinal Fatty Acid Binding Protein (I-FABP), Interleukin-8 (IL-8), and Tissue Factor (TF) Concentrations

Venous blood was drawn from the antecubital region before and at the end of the heat exposure. Participants were rested for at least 30 min prior to blood draws for the stabilization of plasma volume. Hematocrit (microcapillary technique) and hemoglobin (ABL90 Flex, Radiometer, Brønshøj, Denmark) were measured and used to calculate relative changes in plasma volume using the Dill and Costill [32] equation. Blood samples were centrifuged to isolate plasma and aliquots of plasma were stored at −80 °C.

Systemic I-FABP, IL-8, and TF concentrations were assessed at baseline and immediately following the 3-h heat exposure (before exiting the environmental chamber) using enzyme-linked immunosorbent assays (ELISA) (R&D Systems, Minneapolis, MN, USA), according to manufacturers’ instructions. These markers were used to assess enterocyte damage, systemic inflammation, and blood coagulation, respectively. Samples obtained at the end of the heat exposure were corrected for the change in plasma volume [32].

### 2.8. Statistical Analyses

**Figure 2:**
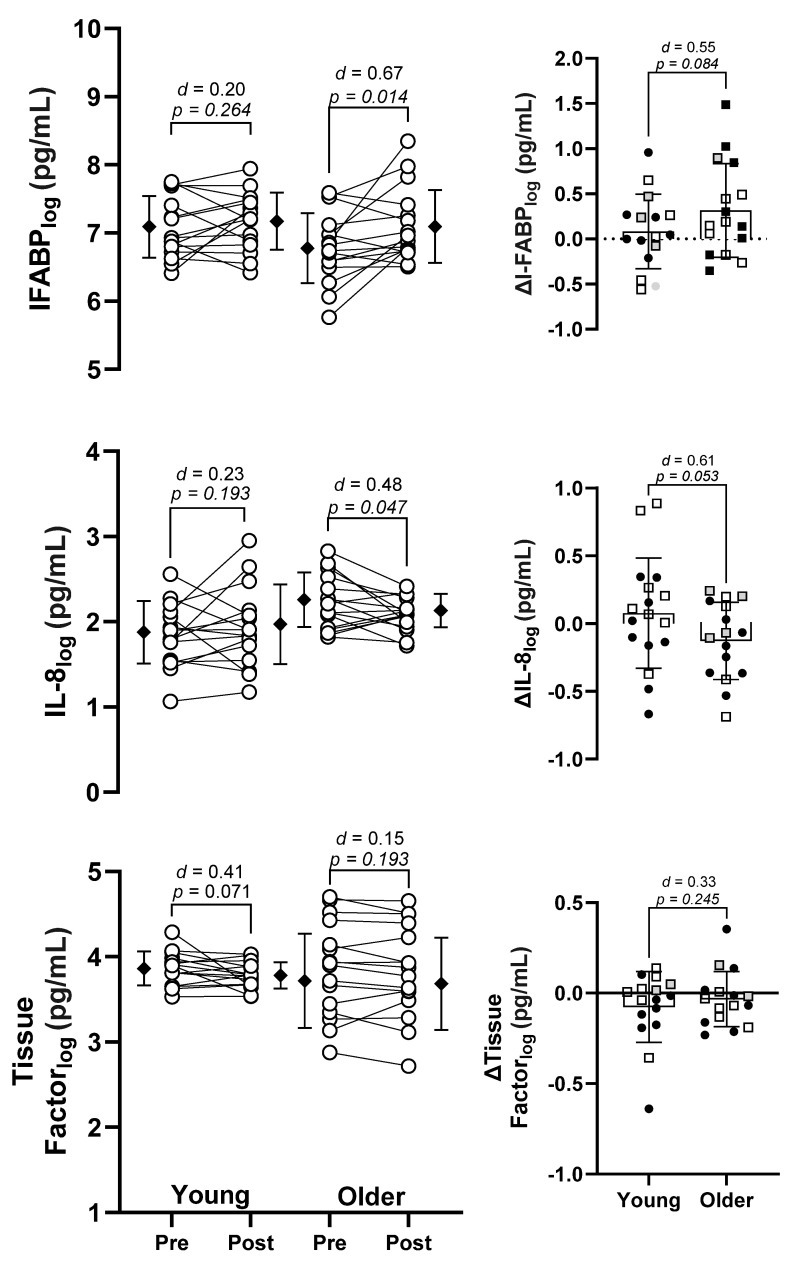
Age-related changes in log-transformed plasma intestinal fatty acid binding protein (IFABP_log_), interleukin–8 (IL-8_log_), and Tissue Factor before and after a 3-h simulated heat wave. Filled diamonds show the mean and standard deviation. Right-hand panels show the difference from pre to post heat exposure. Filled circles represent males; open squares represent females.

**Figure 3:**
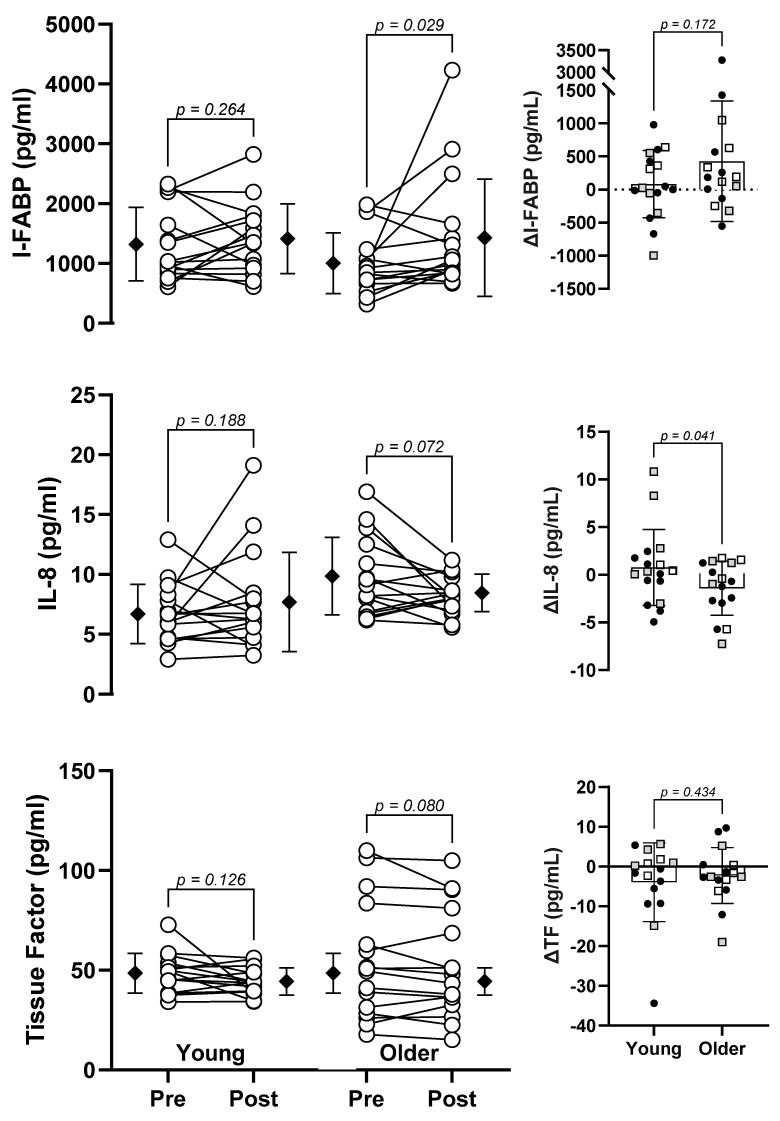
Absolute values for plasma intestinal fatty acid binding protein (IFABP), interleukin-8 (IL-8), and Tissue Factor before and after a 3-h simulated extreme heat event in young and older adults. Data analyzed by non-parametric methods. Filled diamonds show the mean and standard deviation. Filled circles represent males; open squares represent females.

Statistical analyses were performed using GraphPad Prism version 9. A Shapiro–Wilk test showed that I-FABP, IL-8, and TF were not normally distributed. Once log-transformed, these variables were normally distributed and thus were analyzed using parametric statistics (see Figure 2). That said, absolute values for I-FABP, IL-8, and TF are shown in Table 2 and Figure 3 to aid readability and comparison with previous studies. Statistical analyses of these absolute values were conducted using either a Wilcoxon test (for within-subject comparisons) or a Mann–Whitney test (for between-subject comparisons). Internal (core) body temperature, heart rate, and mean arterial pressure were all normally distributed and are thus reported as mean and standard deviation. Paired *t*-tests were used to compare each variable pre and post heat exposure within the young and older groups. An independent samples *t*-test was used to compare if the magnitude of the change in each variable to the heatwave simulation was different between age groups. Cohen’s d was utilized as an additional measure of the difference between pre and post heat exposure using the repeated-measures version of Cohen’s d that factors in the correlation between time points [36]. The classic version of Cohen’s d was used to determine the effect size for the change in each variable between age groups. As an exploratory analysis, correlation analysis was performed to determine whether the change in I-FABP_log_ pre to post heat exposure was related to the change in core temperature, heart rate, or mean arterial pressure. Log-transformed data are reported as mean ± SD and 95% confidence interval. Consistent with prior work from our lab [37], we did not create a dichotomous line of significance/nonsignificance [38,39]. However, when *p* values were below 0.10, we considered that value, along with the physiological relevance for each variable, to conclude the result of a given comparison. Finally, we report the effect size to aid with interpretation.

## 3. Results

### 3.1. Core Body Temperature

In young adults, the core temperature increased from pre (37.1 ± 0.3° C) to post (37.8 ± 0.2° C) heat exposure (*p* < 0.001, *d* = 2.57), while the core temperature increased from pre (36.8 ± 0.3° C) to post (38.2 ± 0.4° C) heat exposure (*p* < 0.001, *d* = 3.30) in the older group. The magnitude of the increase in core body temperature was greater in the older group (*p* < 0.001, *d* = 1.5). A comprehensive assessment and comparison of the thermoregulatory responses between young and older adults is available in our companion manuscript [36].

Descriptive data and non-parametric analysis for absolute I-FABP, IL-8, and TF are shown in Table 2.

### 3.2. Log Intestinal Fatty Acid Binding Protein (I-FABP_log_)

In young adults, I-FABP_log_ did not change from pre (7.12 ± 0.52 pg/mL) to post (7.19 ± 0.46 pg/mL) heat exposure (*p* = 0.264, *d* = 0.20). In older adults, I-FABP_log_ increased from pre (6.79 ± 0.51 pg/mL) to post (7.11 ± 0.54 pg/mL) heat exposure (*p* = 0.014, *d* = 0.67). There was a moderate age difference for the magnitude of the change in I-FABP_log_ from pre to post heat exposure between groups (*p* = 0.084, *d* = 0.55). These results are shown in Figure 2.

### 3.3. Log Interleukin-8 (IL-8_log_)

In young adults, IL-8_log_ did not change from pre (1.84 ± 0.37 pg/mL) to post (1.93 ± 0.47 pg/mL) heat exposure (*p* = 0.193, *d* = 0.23). In older adults, IL-8_log_ decreased from pre (2.22 ± 0.32 pg/mL) to post (2.09 ± 0.20 pg/mL) heat exposure (*p* = 0.047, *d* = 0.48). There was a moderate age difference for the magnitude of the change in IL-8_log_ from pre to post heat exposure between groups (*p* = 0.053, *d* = 0.61). These results are shown in Figure 2.

### 3.4. Log Tissue Factor (TF_log_)

In young adults, TF_log_ decreased from pre (3.86 ± 0.20 pg/mL) to post (3.78 ± 0.15 pg/mL) heat exposure (*p* = 0.070, *d* = 0.41). In older adults, TF_log_ did not change from pre (3.86 ± 0.54 pg/mL) to post (3.83 ± 0.54 pg/mL) heat exposure (*p* = 0.193, *d* = 0.15). There was a small effect for the magnitude of the change in TF_log_ from pre to post heat exposure between groups (*p* = 0.245, *d* = 0.33). These results are shown in Figure 2.

Absolute values of I-FABP, IL-8, and Tissue Factor are shown in Figure 3. Non-parametric analysis of the absolute data is also presented within the figure.

### 3.5. Correlation Analysis

**Figure 4:**
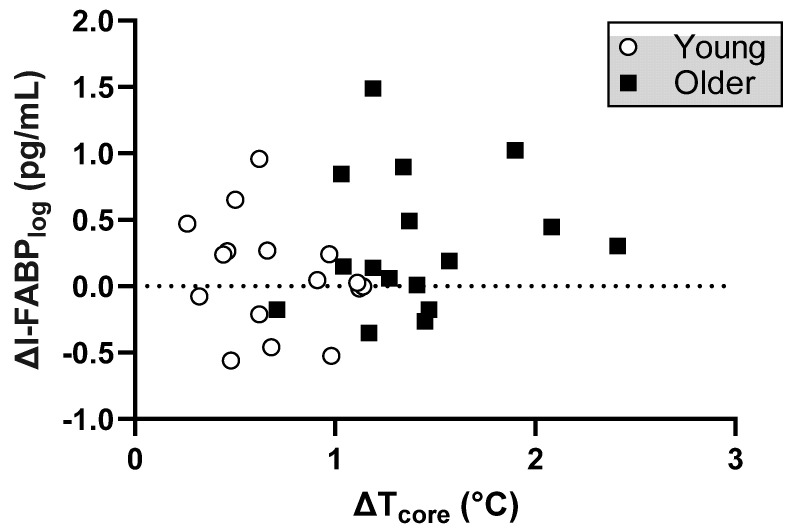
No correlation between the change in core temperature (ΔT_core_) and the change in log-transformed intestinal fatty acid binding protein (ΔI-FABP_log_) with both groups combined.

As shown in Figure 4, there was no correlation between the change in I-FABP_log_ and the increase in core temperature in young (*p* = 0.361) and older (*p* = 0.586) subjects, or when data for both groups were pooled (*p* = 0.318). Furthermore, no correlation was found when comparing end values of I-FABP with end core temperature (*p* > 0.05).

## 4. Discussion

This preliminary study aimed to test the hypothesis that, in response to a simulated heat wave, markers of enterocyte damage would be greater in older adults, compared with younger adults. Our experimental hypothesis was accepted, as plasma I-FABP, a marker of enterocyte damage, increased in the older cohort, but not the younger cohort. A secondary aim was to test the hypothesis that plasma markers of systemic inflammation (IL-8) and coagulation (TF) would be greater in the older cohort compared with the younger cohort. This hypothesis was not accepted, in that the simulated heat wave did not cause appreciable changes in either of these markers in either group. A final aim was to explore whether any changes in I-FABP were associated with changes in core body temperature. Correlation analysis showed that the change in core temperature was not associated with the change in I-FABP.

To our knowledge, this is the first study to examine whether aging changes the markers of gastrointestinal barrier damage during exposure to heat stress. Figure 2 and Figure 3 show the log-transformed and absolute values for each variable, respectively. Importantly, there was a significant increase in the older cohort, but not the young cohort, for I-FABP, regardless of which data set was analyzed. It is also interesting to note that baseline levels of I-FABP were lower in the older group. This observation corroborates animal studies that report a decrease epithelial turnover rate in old mice, compared with young mice, potentially due to a reduction in epithelial stem cell function [40,41]. Although our data are preliminary, we provide evidence that older individuals may suffer negative health effects during heat waves due to a loss of gastrointestinal barrier integrity. For example, if gastrointestinal barrier function is decreased during heat stress, microbial translocation can occur, invoking a systemic inflammatory response that can be fatal [10]. There are several lines of evidence supporting this pathophysiological hypothesis in the clinical setting. First, older individuals admitted to the ICU due to heat stroke showed similar pro-inflammatory responses to normothermic, age-matched septic patients [26]. In that study, the degree of inflammation was strongly associated with the risk of mortality, primarily owing to the development of disseminated intravascular coagulation. Moreover, of those patients admitted to the ICU due to heat stroke during the 1995 Chicago heat wave, ~60% showed evidence of bacteremia [42]. With more sensitive techniques, such as ELISA or whole-blood 16S sequencing, it is possible that systemic inflammation and/or microbial translocation would have been detected in a greater number of these patients. Since I-FABP was elevated in older people but not younger people during our simulated heat wave, it is likely that older individuals are more susceptible to heat-induced gastrointestinal barrier damage and, consequently, microbial translocation.

Reducing microbial translocation by preserving GI barrier integrity is likely to increase physiological tolerance to a given heat exposure. This view is supported by evidence from animals and humans. First, non-human primates administered with an anti-endotoxin antibody demonstrate profound increases in survival rate during severe hyperthermia [24,25,43]. Second, humans with high aerobic fitness show greater tolerance to hyperthermia than their unfit counterparts [44]. While the primary reason for these differences is likely due to elevated cardiovascular stress in unfit individuals, fitter individuals show reduced microbial translocation during matched increases in core temperature [45]. Since older individuals are typically less fit than younger individuals [46], lower aerobic fitness may also contribute to greater enterocyte damage during heat exposure. The separate and combined effects of age and fitness on enterocyte damage and microbial translocation during heat stress therefore warrant further investigation.

The variability in the I-FABP response to heat could not be explained by core temperature in our study. Thus, elevations in I-FABP may not be linked to enterocyte temperature directly, at least within the range tested. Instead, it is possible that the extent of heat-induced redistribution of blood flow from the intestines increases I-FABP, a response that is likely highly variable between subjects [14,16,47]. This high degree of variability between subjects likely explains the lack of direct correlation with core temperature in our study. Data from anesthetized rats suggest that the oxidative stress pathway is directly responsible for enterocyte damage and microbial translocation during hyperthermia [19]. In that study, elevating the core temperature from 37 to 41.5 °C decreased mesenteric artery flow by 40%, and that ischemia (reduced oxygen supply) stimulated the production of free radicals. With hyperthermia, portal endotoxin concentrations doubled, yet pretreatment with allopurinol (a xanthine oxidase antagonist) eliminated the increase in portal endotoxins and increased survival. Moreover, further decreasing splanchnic blood flow with L-NAME further increases portal endotoxin concentrations and the likelihood of fatality. Finally, exercise in hypoxia increases I-FABP to a greater extent than in normoxic conditions [21,22]. Taken together, it is possible that free radical-induced damage to enterocytes, perhaps associated with heat-induced reductions in splanchnic blood flow, is an early mediator of heat stroke.

Unlike I-FABP, there was no heat-induced increase in plasma IL-8 or Tissue Factor in either group (Figure 2). Increased concentrations of IL-8 are indicative of a pro-inflammatory response associated with microbial translocation or exogenous infection. IL-8 is a chemokine produced by several cell types to attract neutrophils, basophils, and T-cells to the site of infection, and is critically involved in the early inflammatory response to infection [26,48]. A previous study using sauna-induced dehydration showed that IL-8 was significantly and consistently elevated post heat exposure [34]. The reason why IL-8 did not similarly increase in our study could be due to us providing fluid to the participants throughout the protocol. Osmotic stress induces an inflammatory response in Caco-2 cells [49], which likely explains the increase in IL-8 in that work, and no increase in IL-8 in our work. Tissue Factor concentrations are increased by pro-inflammatory cytokines to initiate the blood coagulation cascade. It is well accepted that sepsis (originating from endogenous or exogenous microorganisms) can cause coagulation defects resulting in a sustained hypercoagulable state, potentially leading to disseminated intravascular coagulation [50]. While each of these markers are implicated in the pathophysiology of heat stroke [51,52], in our study, it is likely that the magnitude of the heat strain imposed was insufficient to initiate these responses, despite elevating I-FABP in the older group. The decrease in IL-8 reported in older individuals cannot yet be explained.

Several regulatory mechanisms that minimize elevations in gastrointestinal permeability are disrupted with aging. For example, aging can promote microbiota dysbiosis, inducing a pro-inflammatory state within the gut [29], decrease mucin secretion [30], decrease Paneth cell function [31], and decrease microbial neutralization in the portal circulation and liver [53]. Further, the survival outcomes following severe sepsis are reduced with aging [54], which may be linked to a dysregulated immune response (immunosenesence) to infection. At baseline, older adults also have reduced splanchnic/intestinal blood flow [18], which may be detrimental to gastrointestinal barrier integrity by decreasing the oxygen supply to enterocytes. The above evidence, combined with our preliminary data showing that the change in I-FABP is greater in the older cohort, suggests that older people may be more at risk of microbial translocation during heat waves. This view challenges the current dogma suggesting that the majority of heat wave-related deaths are primarily cardiovascular related (i.e., heart attack, stroke, etc.). Given that (1) reduced splanchnic blood flow and subsequent bacteremia initiate heat stroke in animal models [19,20,25,51,52,55,56], (2) gastrointestinal barrier function is reduced with aging [29,30,31,53], and (3) heat exposure elevated I-FABP in older people but not younger people in our study, future work investigating if older individuals are susceptible to this pathophysiology during heat waves is justified.

## 5. Limitations

There are two primary limitations that should be considered for our study. First, the assessment of I-FABP was not a primary aim of the wider project, hence we lacked dietary controls that should ideally be considered when assessing I-FABP. Although we restricted alcohol and exercise for 24 h before the trial, we did not strictly control diet, allowing participants to consume a light breakfast of their choosing prior to arrival. A second limitation was that we did not directly measure gastrointestinal permeability. Gastrointestinal permeability can be assessed using orally ingested sugar probes and analyzing their urinary concentrations several hours after ingestion. While plasma I-FABP is an accepted marker of enterocyte damage, it does not always correlate well with direct permeability measures, and it is therefore unknown if permeability was elevated in the older group in our study. Finally, due to the known thermoregulatory impairments associated with aging (i.e., reduced sweating), the core temperature elevation was greater in the older group compared with the young group. While the core temperature itself was not correlated with I-FABP, it is unknown whether downstream cardiovascular responses to hyperthermia (perhaps greater reductions in splanchnic blood flow) mediated the elevated I-FABP response in the older population in our study. Importantly, the core temperature response was intentionally not matched between groups, reflecting the core temperature patterns that are likely to occur during a real-world heat wave. Despite these limitations, the present work supports the need to further understand the impact of aging on gastrointestinal permeability during extreme heat exposure.

## 6. Conclusions

In conclusion, our preliminary data show that I-FABP, a marker of enterocyte damage, is elevated in older people, but not younger people, during a simulated heat wave. Furthermore, we show that the magnitude of the change in I-FABP is not related to the increase in internal core temperature during a simulated heat wave challenge.

## Figures and Tables

**Table 1:** Subject characteristics. Data are shown as mean (SD) and range.

	Young (8M/8F)		Older (8M/8F)		*p* Value
**Age (years)**	30 (5)	21 to 39	70 (4)	65 to 76	-
**Height (cm)**	171 (8)	161 to 190	169 (9)	153 to 186	0.620
**Mass (kg)**	70.2 (10.3)	60.2 to 97.9	75.3 (12.1)	57.3 to 94.0	0.197
**BSA (m^−2^)**	1.81 (0.16)	1.65 to 2.20	1.86 (0.19)	1.55 to 2.19	0.447
BMI (kg/m^−2^) *	24 (3)	20 to 29	26 (2)	21 to 30	0.041
Fat (%) *	29 (8)	14 to 40	37 (7)	23 to 48	0.006
**Baseline USG**	1.009 (0.005)	1.000 to 1.019	1.012 (0.006)	1.003 to 1.023	0.103
**Baseline MAP (mmHg)**	91 (7)	77 to 101	91 (5)	82 to 100	0.969

BSA, Body surface area; BMI, Body mass index; USG, Urine specific gravity; MAP, Mean arterial pressure; * different between age groups (*p* < 0.05).

**Table 2:** Mean and standard deviation for absolute concentrations of blood markers, and their change from pre to post heat exposure.

	Young		Δ	Older		Δ
	Pre	Post		Pre	Post	
**I-FABP** (pg/mL)	1320 ± 615	1410 ± 584	89 ± 522	1002 ± 507	1429 ± 980	427 ± 910
**IL-8** (pg/mL) *	6.67 ± 2.48	7.68 ± 4.15	1.01 ± 3.98	9.64 ± 3.28	8.22 ± 1.59	−1.42 ± 2.85
**TF** (pg/mL)	48.5 ± 9.9	44.4 ± 6.8	−4.13 ± 16.2	54.6 ± 29.4	52.4 ± 27.0	−2.3 ± 7.0

I-FABP, Intestinal fatty acid binding protein; IL-8, Interleukin-8; TF. Tissue Factor; Δ, change from pre to post. * = IL-8 baseline different between age groups (*p* = 0.006).

## Data Availability

The data presented in this study are available on request from the corresponding author. The data are not publicly available due to data privacy issues.
